# Spatial differences in thermal comfort in summer in coastal areas: A study on Dalian, China

**DOI:** 10.3389/fpubh.2022.1024757

**Published:** 2022-10-11

**Authors:** Hongchi Zhang, Fei Guo, Kaitong Liu, Jie Wang, Jing Dong, Peisheng Zhu

**Affiliations:** School of Architecture and Fine Art, Dalian University of Technology, Dalian, China

**Keywords:** coastal city, outdoor thermal comfort, coastal park, commercial street, universal thermal climate index

## Abstract

Thermal comfort is an important indicator for evaluating the environment of urban public space, and appropriate thermal comfort can effectively prolong the duration of outdoor activities. In the existing studies, there is a lack of thermal comfort comparison between hot spots and cold spots. In this study, we selected the coastal city of Dalian in China as our study area and conducted field investigations on the thermal comfort of two landmark resorts, namely, a downtown commercial street and coastal leisure park. The study was conducted on typical summer days and consisted of interviewing several residents to understand their thermal comfort requirements. We investigated the thermal expectations of the interviewees through meteorological measurements and questionnaires. The universal thermal climate index (UTCI) was used to determine the thermal benchmarks of the on-site subjects. The results indicated that (1) globe temperature and air temperature were the most important factors that affected thermal comfort, followed by relative humidity and wind speed in summer daytime. (2) Shaded spaces are more comfortable than open spaces, and tree shade is preferred over artificial shade in coastal park. (3) The neutral UTCI (NUTCI) of the respondents were 24.1°C (coastal park) and 26.0°C (commercial street); the neutral UTCI ranges (NUTCIR) were 20.8–27.4°C (coastal park) and 23.3–28.7°C (commercial street). (4) The upper thermal acceptable range limits of the coastal park and commercial street were 30.2 and 32.1°C, respectively, which were substantially higher than the upper NUTCIR limit, indicating that the residents in Dalian were well-adapted to hot weather. The results can provide a good reference for determining ideal design strategies to optimize the thermal environment of urban outdoor recreation spaces in summers and improve the quality of life in coastal cities.

## Highlights

- We conducted field investigations on thermal comfort in two areas in Dalian, China.
- Survey was conducted (>800 respondents) and various indexes were analyzed.
- Respondents felt more comfortable in shaded areas, especially in the shade of trees.
- People in commercial street were more adapted to high temperatures, and people in coastal area preferred cooler environments.
- The residents in Dalian (coastal city) were well-adapted to hot weather.

## Introduction

Extreme hot weather and heat waves caused by global warming, along with urban heat islands caused by urbanization, have greatly affected the livability of urban regions (1–4). A comfortable outdoor environment can not only prolong the duration of outdoor activities and reduce the use of air conditioning, thus, reducing building energy consumption (5), but also optimize urban space utilization and enhance outdoor activities, social solidarity (6, 7), public health (8, 9), and tourism (10). Relevant studies conducted in different climate zones have shown that the regional thermal benchmarks of outdoor thermal comfort (OTC) need to be calibrated, due to the possible differences in their thermal history (11), social culture (12, 13), and thermal adaptation of local residents (14), along with other factors. Therefore, determining the outdoor thermal benchmark of a region can help urban planners to design and plan open urban spaces effectively to enhance outdoor thermal comfort and improve urban microclimate (15).

OTC has been extensively studied in continents other than Antarctica (16, 17), focusing on dry and hot (18), severe cold (19), humid and hot (20), and subtropical climate zones (21), and some cities have been investigated repeatedly. Investigating *in-situ* thermal conditions vs. subjective thermal perception has become a routine method for assessing human thermal perception in different climate zones (22–24). However, due to different measurement procedures (location, season, time) and measurement tools, the same city may show different comfort ranges by using the same analysis methods, e.g., the UTCI “no thermal stress” range in Xi'an was defined as 18.0–29.1°C (11) and as 15.8–28.5°C (25), and the PET neutral range in Harbin is defined as 8.5–26.8°C (26) and as 13.0–21.0°C (27). In addition, the OTC of different locations in a city may vary significantly depending on urban morphology or land cover factors (28–31), such as sky view factor (SVF), building height, compactness, street orientation, vegetation, and albedo (32–37). Accordingly, the above issues should be fully considered in OTC surveys and analyses.

Most of the studies investigated one type of urban outdoor environment e.g. squares (38), parks (36), campuses (26, 39), scenic areas (40), streets (27, 41), etc. However, the literature (17) suggests that both hot spots (squares, main streets) and cold spots (parks, water bodies) should be considered when discussing the OTC of a city. Some studies fully compared OTC in different areas of the city, but they are similar in one type, such as the study of Umeå compared city park and university park (42), the study of Melbourne compared three university campuses (43), and the study of Victoria selecting 11 streets with different orientation and geometry (41). There are also some studies that fully considered multiple space types in a city, for example the study (44) that compared human thermal perception outdoors in summer in Singapore and Changsha by selecting 13 and 17 outdoor measurement points, including parks, squares, commercial streets and university campus respectively, but did not compare the OTC of each type of space, however, no comparison was made among the types of space thermal comfort. The study in Tel Aviv (45) selected city parks, city squares and wide streets in low-density neighborhoods for a 4-year variability study and compared them with different climate zones, with the only regret that only one measurement point was available for each space type. Although a large number of studies are currently for inland cities (16), OTC studies in coastal cities have also been conducted extensively, including Shanghai (46), Hong Kong (21), and Nagoya (47) in addition to the above cities, but comparative studies of OTC in open coastal areas and within high-density urban areas are still deficient.

In summer coastal cities, people generally stay outdoors for longer durations, and the most significant spaces for leisure activities are coastal parks and commercial streets near the city center, and there are crucial differences between the spatial characteristics of urban centers and those of the parks near the coastline. Due to limited land resource and high levels of development, commercial streets in urban centers generally portray high-density and high-rise urban form patterns, with lower greening ratio, in contrast to the low density and high greening ratio of coastal parks (48). In addition, the climate conditions in low-density coastal areas and the high-density neighborhood are generally quite different (49), for instance, coastal areas may feature higher wind speed and solar radiation, besides, the heat island intensity as well as the temperature in commercial areas is higher (50).

Most studies have confirmed that physical factors centered on microclimate parameters have the greatest influence on human thermal perception, and among the four microclimate variables of air temperature (*T*_*a*_), wind speed (*V*_*a*_), relative humidity (RH), and globe temperature (*T*_*g*_), many studies have confirmed that *T*_*a*_ is the most important parameter for OTC (51, 52), but there are few low-cost methods to reduce the local *T*_*a*_ (53). Therefore, a more effective approach in creating thermally comfortable open urban spaces is to control thermal radiation and wind speed (16). Furthermore, previous studies have confirmed that wind speed and the difference in radiant temperature have equally significant effects on thermal comfort (54). Therefore, it is important to identify the factors that influence the spatial thermal comfort in coastal cities to optimize the designs of urban spaces.

In this study, the universal thermal climate index (UTCI) was used to study the OTC in the coastal city of Dalian in summer. Two of the most famous resort sites in the city, the Xi'an Road (commercial street) and Xinghai Park (coastal park), known to host frequent outdoor social activities in summer, were selected as the study areas. We selected six measurement points for each study site to conduct the meteorological measurements and questionnaire surveys and ensured that the spatial types were rich enough to reflect the effects of spatial differences on human thermal comfort. The objectives of this study were to (1) analyze the main meteorological elements that affect the OTC in coastal cities in summer; (2) compare the thermal comfort expectations of different areas and types of leisure spaces in coastal cities, including thermal sensation, thermal comfort, and thermal preference; and (3) establish outdoor thermal benchmarks (e.g., neutral temperature, neutral temperature range, and thermal acceptable range) for littoral regions and business districts in coastal cities and discuss their spatial differences. The results of the study may provide theoretical evidence that can improve the quality of open spaces in summer coastal cities, enhance urban design strategies, and enable governance at a finer spatial scale.

## Materials and methods

### Study area

**Figure 1:**
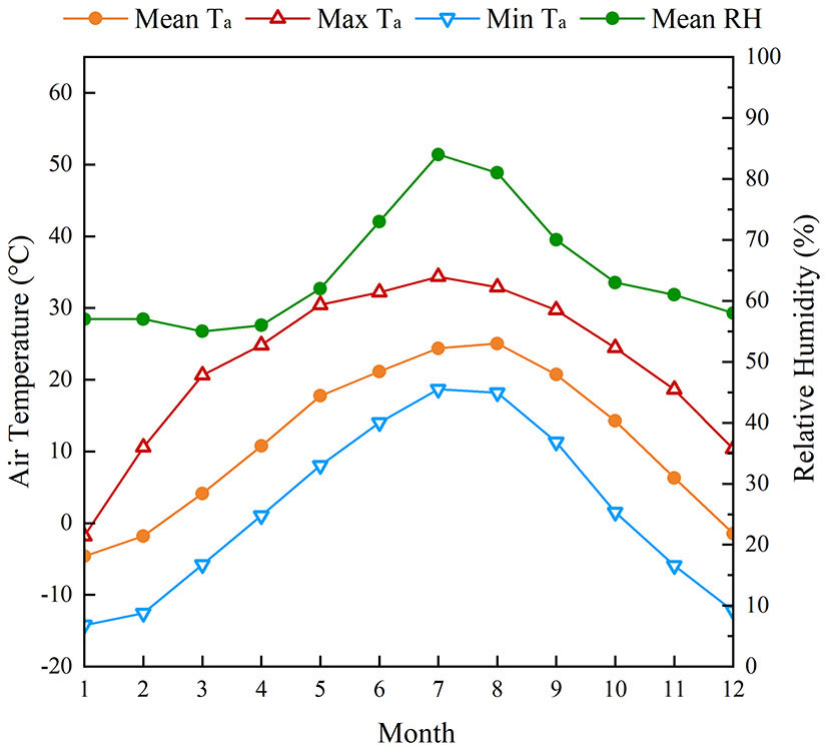
Monthly mean, maximum, and minimum air temperature (*T*_*a*_) and mean relative humidity (RH) in Dalian, China, from 2011 to 2021 (56).

Dalian (121°6′ E, 38°9′ N), a famous coastal tourist city in China, is located on the Liaodong Peninsula and surrounded by the sea on three sides, making it an ideal habitable coastal city that can provide an ideal environment to study the OTC in coastal cities. The climate in Dalian is relatively pleasant in all seasons, with no severe cold in winters and pleasant summers. According to the Köppen climate classification (55), it is located in a temperate monsoon climate (Dwa, D: cold, w: dry winter, a: hot summer), with maritime characteristics. According to meteorological data from 2011 to 2021, the highest monthly mean temperature occurred in August (25.0°C), and the maximum monthly mean temperature occurred in July 34.4°C. The hottest days in summer in Dalian mainly occur from late July to early August. The lowest monthly mean temperature occurred in January (-4.6°C), with a minimum temperature of −14.2°C. The annual average RH was generally between 51 and 80% (56) (Figure 1).

**Figure 2:**
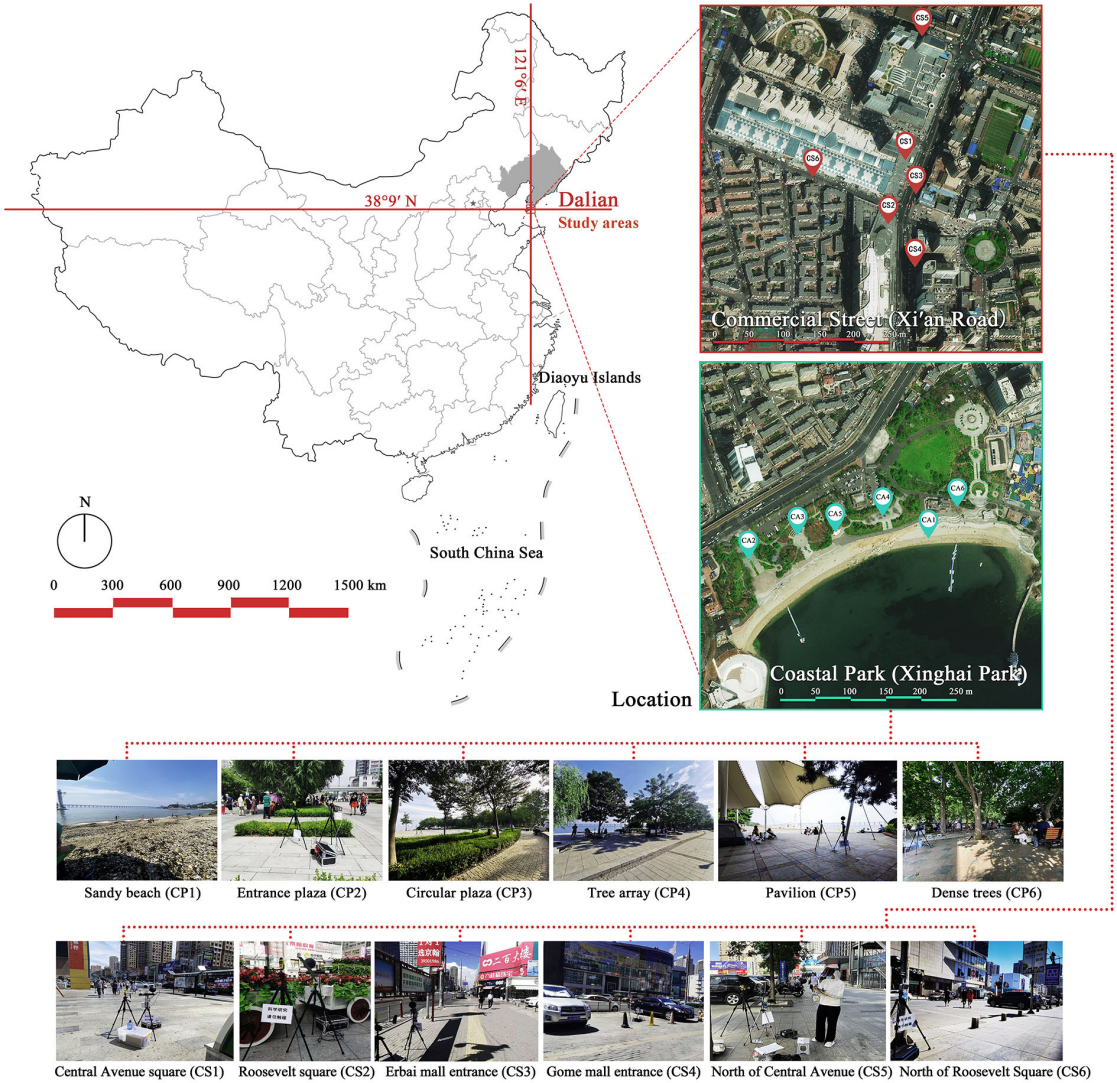
Study areas considered in this study and the six measurement points for the Xinghai Park and Xi'an Road (CP1–CP6 and CS1–CS6, respectively).

Field tests were conducted in a famous coastal park (Xinghai Park) and commercial street (Xi'an Road) in Dalian, China. As the two space types differed greatly, with Xinghai Park being more open and Xi'an Road having a higher building density, we selected six measurement points each in both the areas (CP1–CP6, CS1–CS6, respectively) and compared the differences in the factors that influenced the thermal comfort in these areas, while considering the openness and radiation orientation of the space and their shaded regions (57) (Figure 2).

The *T*_*a*_ in urban spaces within a radius of 10–150 m from the center were influenced by the surrounding environment (58). Therefore, the composition of the landscape within the range of 10 m was measured from the physical midpoint of the 12 measurement points (CP1–CP6 and CS1–CS6) (40). Fisheye photographs of the measurement points were captured and input into the RayMan software to calculate their corresponding SVF values (Table 1).

**Table 1:** Characteristics of measuring points of Xinghai park and Xi'an road.

**Coastal park (Xinghai park)**			**Commercial street (Xi'an road)**		
**Test location**	**Measuring-point environment**	**Fisheye photo**	**Test location**	**Measuring-point environment**	**Fisheye photo**
CP1 (Beach)	Close to the sea and backed by tableland. Full of coarse sand and pebbles.	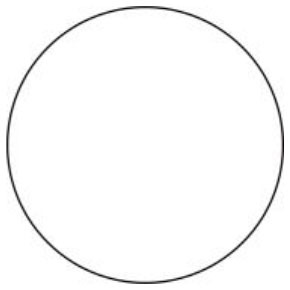 SVF = 1	CS1 (Central avenue square)	The plaza in front of the mall is paved with marble. The square was open and unobstructed.	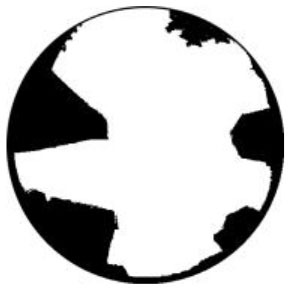 SVF = 0.718
CP2 (Entrance plaza)	Connects the beach to the city road, with dense vegetation on both sides and flower beds in between, marble groud.	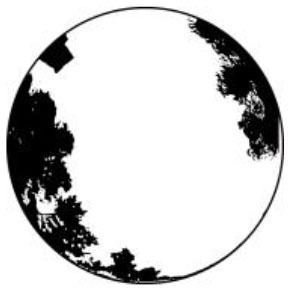 SVF = 0.793	CS2 (Roosevelt square)	The plaza in front of the mall is paved with marble. The plaza was empty and somewhat sheltered by buildings on the south side.	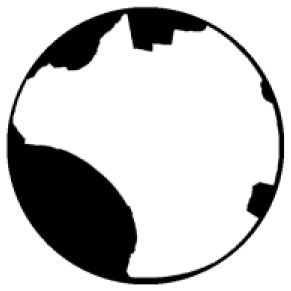 SVF = 0.704
CP3 (Circular plaza)	Semi-enclosed, open to the marine area, backed by a circular terrace bench and planted with rows of trees. Marble groud.	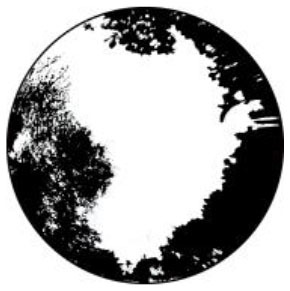 SVF = 0.579	CS3 (Erbai Mall entrance)	The north-south streets are covered with permeable brick, screened by multi-story building to the southeast.	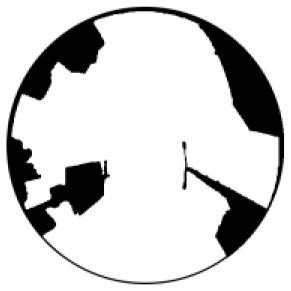 SVF = 0.789
CP4 (Tree array)	The space is a resting space formed by a combination of large and small tree beds, with granite slate flooring.	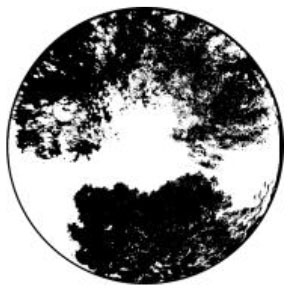 SVF = 0.335	CS4 (Gome Mall entrance)	The north-south streets are covered with permeable brick, screened by high-rise building on east side.	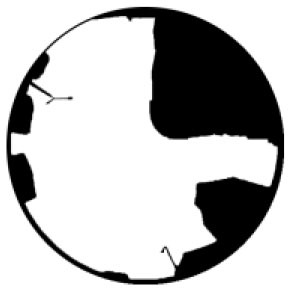 SVF = 0.644
CP5 (Pavilion)	Seaside tensioned membrane structures for pavilions, marble groud.	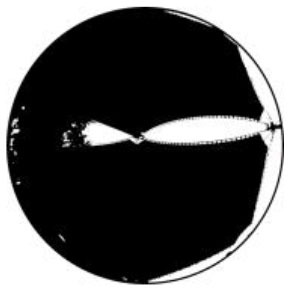 SVF = 0.097	CS5 (North of Central Avenue)	East–west street have marble floors, with commercial buildings on the south side and more trees in the street	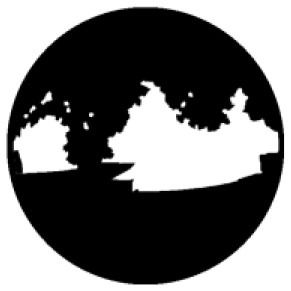 SVF = 0.240
CP6 (Dense trees)	Dense forest area. Dense woods and buildings creating large shade. Marble groud.	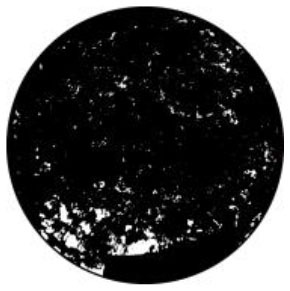 SVF = 0.008	CS6 (North of Roosevelt Square)	East–west street have marble floors, with multistory building on south side, perimeter parking, and no vegetation.	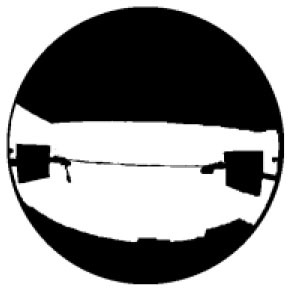 SVF = 0.426

### Physical measurements

In this study, we selected the typical hot weather days in Dalian city in summer to conduct the field tests; the measurement period was from 09:30 to 17:30 h, which was the peak time for outdoor activities. The tests were conducted on July 24, 25, 26, and 28 and August 4 in 2021 (a total of 5 days). The meteorological parameters, namely, *T*_*a*_, *RH, V*_*a*_ and *T*_*g*_, were recorded every min. The measuring instruments used for this purpose and the parameters considered in this study are listed in Table 2, all in accordance with the ISO 7,726 standard (59).

**Table 2:** Parameters considered in this study and the instruments used to measure them.

**Parameter**	**Instrument**	**Range**	**Accuracy**
Air Temperature (*T_*a*_*)	HOBO, MX2301	−40–0°C 0–70°C	±0.25°C ±0.2°C
Relative humidity (RH)	HOBO, MX2301	0–10% 10–90% 90–100%	±5% ±2.5% ±5%
Air Velocity (*V_*a*_*)	Kestrel 5,500	0.1–9.99 m/s 10.0–20.0 m/s	+ (0.05 m/s + 5% readout) + (5% readout)
Globe Temperature (*T_*g*_*)	JTR10 WBGT	5–120°C	± 0.5°C

According to American Society of Heating Refrigerating and Air-conditioning Engineers (ASHRAE) standard 55 (60) and some field studies (61, 62), there is no significant difference in the measured meteorological parameters above 0.6 m (head level), above 1.1 m (abdomen level) and above 1.7 m (ankle level). So, the sensors were set at 1.5 m above ground level in the formal field survey. We calculated the mean radiant temperature (*T*_*mrt*_) using the ISO 7,726 standard, as shown in Equation (1) below:


(1) $$\begin{array}{l} T_{mrt}={\left[{\left(T_{g}+273\right)}^{4}+\frac{1.10\times 10^{8}{V_{a}}^{0.6}}{\varepsilon D^{0.4}}\left(T_{g}-T_{a}\right)\right]}^{\frac{1}{4}}-273 \end{array}$$

where *D* is the globe diameter (*D* = 0.15 m in this study), and ε is the emissivity (ε = 0.95 for a black globe).

### Questionnaire surveys

The questionnaire used for the study was divided into two parts (Appendix A, Figure A1). The first section contained basic information about the interviewee, including gender, age, height, weight, clothing, thermal history, and purpose and intensity of activity. We used the ASHRAE standard 55 (60) to determine the interviewees' thermal resistance of clothing and their metabolic rate. We measured the field meteorological data in conjunction with the questionnaire, to ensure accuracy, the measurements were conducted within 3 m of the position of the measuring instruments.

The second part consisted of thermal sensation information, including votes for the thermal sensation, preference, and acceptability and adaptive behavior of the respondents. One of these votes, the thermal sensation vote (TSV), was quantified using a seven-point scale (−3, cold; −2, cool; −1, slightly cool; zero, neutral; one, slightly warm; two, warm; three, hot), based on the ASHRAE standard. The thermal preference vote (TPV) was measured using a three-point scale (−1, lower/weaker; zero, no change; one, higher/stronger). The thermal acceptability vote (TAV) was measured using a four-point scale (−2, completely unacceptable; −1, unacceptable; one, acceptable; two, completely acceptable).

### Thermal comfort index

There is no universal model for studying OTC, and choosing the right model is the key to accurately analyze the OTC of a region (16, 22, 63). Currently, physiological equivalent temperature (64), predicted mean vote (65), UTCI (66), and standard effective temperature (67) are the four most commonly used models.

The application of UTCI in recent OTC assessments has been increasing. The UTCI is defined as the isothermal *T*_*a*_ of a reference condition that can trigger the same dynamic response in a physiological model (66). This index refers to the metric developed by the International Society of Biometeorology based on the concept of equivalent temperature applicable to the major fields of human biometeorology. Furthermore, the index is based on the Fiala's multi-node human physiology and thermal comfort model and simulates the dynamic physiological response by combining a human thermoregulation model with a state-of-the-art clothing model (64). In addition, the UTCI values correspond to heat sensation and stress levels on the UTCI 10 scale (68, 69). Most studies redefined the heat sensation or stress levels for the UTCI according the study site, and many recent studies used the UTCI model and demonstrated that the UTCI thermal index model can be suitable for OTC studies. However, only a few studies have been conducted in the cold regions of China; thus, future studies must consider the applicability of the model in cold regions.

### Data analytical methods

#### Comparative analysis

Comparative analysis is a strategy to determine whether there are comparable differences between two or more different categories. In this study, it was used to analyze the differences in meteorological variables between the points in coastal park and commercial street separately to demonstrate the necessity of each point selected. The meteorological data (*T*_*a*_, *RH, V*_*a*_, *T*_*g*_, *T*_*mrt*_*, UTCI*) were investigated at each of the six measurement points in two study areas.The maximum, minimum, mean and standard deviations (Appendix C) were plotted for meteorological variables of each point during the survey period, but this was not sufficient to show the variability between the measurement points, and due to the very large amount of meteorological data, *post-hoc* multiple testing was required. In this study, Tukey's test was chosen as the *post-hoc* test because the use of this method requires the same sample content for each group. The results are described in section Meteorological parameters and confirm a significant difference between measurement points.

#### Correlation analysis

Correlation analyses were performed to examine the importance of the influence of four conventional meteorological indicators (*T*_*a*_, *RH, V*_*a*_, *T*_*g*_) on thermal sensation vote (TSV) in coastal park and commercial street. Spearman correlation analysis was performed by SPSS (IBM, USA) and the results can be compared with other studies to determine the degree of influence of meteorological variables on human thermal comfort in different areas.

#### Regression analysis

Regression analysis can determine the interdependent quantitative relationship between two or more variables. This study used regression analysis to determine the comfort range, and conducted normality tests on the TSV, TCV, Unacceptable vote, and UTCI sample data, and confirmed that they all met the normal distribution and could be analyzed by regression. In the OTC studies, Linear Regression(LR), Probit Analysis (PA), Ordinal Logistic Regression (OLR), Cubic Regression (CR), Quadratic Fitted Curve (QFC) are universal regression equations (17).

The UTCI range of the mTSV (−0.5, 0.5) was the neutral range (70). The LR method based on bind mTSV that varies between ±0.5, is the most commonly used method to determine the Neutral UTCI range (NUTCIR). The Thermal acceptable range (TAR) based on percentage of acceptability varies between 80%, can modify original UTCI scale in the local climatic zone. The E functions were fitted to the regression models of TAR in this study. The CR and QFC methods are not suitable for this study because our survey time is in the hot summer and there is a lack of cold stress surveys. This E functions was also used in a OTC study of summer campus in Guangdong (71).

## Results

### Descriptive analysis

#### Respondent attributes

A total of 872 volunteers participated in the study, all of whom were local residents, had lived in the city for at least 1 year, and were well-adapted to the local climate. All the participants adjusted their clothing according to their perceptions of temperature change in the city. For the Xinghai Park, 341 volunteers (180 males and 161 females) submitted 337 valid questionnaires. For the Xi'an Road, 531 volunteers (262 males and 269 females) submitted 502 valid questionnaires. The ages of the male and female respondents were 12–91 and 13–82 years, respectively. The subjects included 9.8% children (age < 18 years), 66.5% adults (age 18–60 years), and 23.7% seniors (age >60 years). The proportion of seniors in the coastal area was 41.3%. The average clothing thermal resistance of the respondents was 0.26 clo and 0.32 clo for the Xinghai Park and Xi'an Road, respectively (Appendix B, Table B1).

#### Meteorological parameters

**Figure 3:**
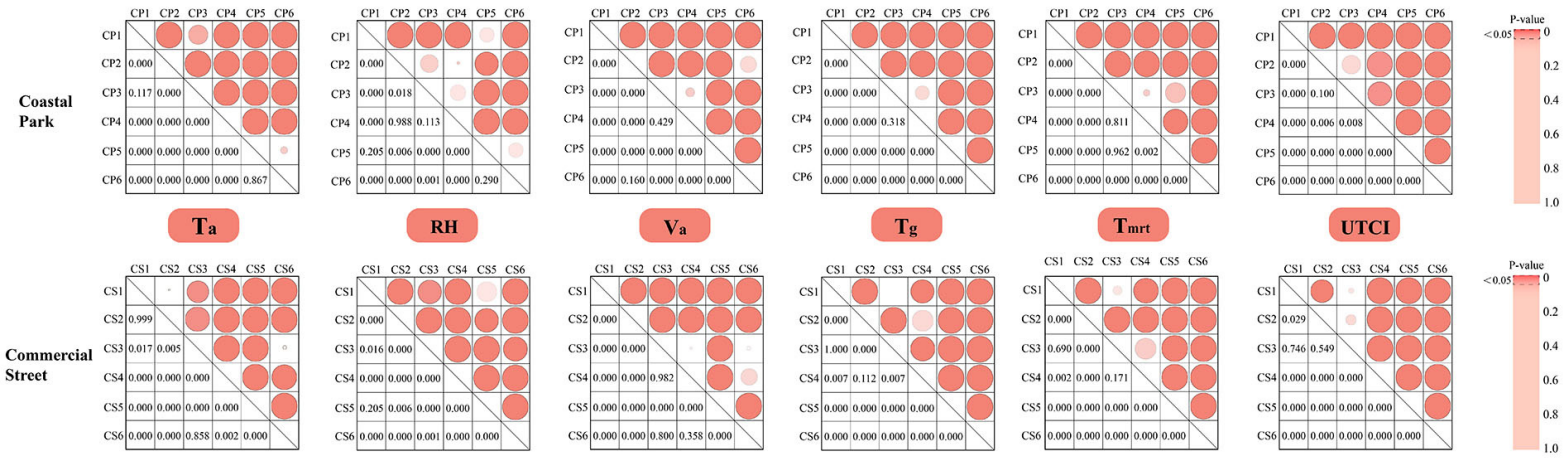
Results of *post-hoc* Tukey's test for different pairs of measurement points for the meteorological variables considered in this study.

The meteorological variables measured in the coastal park and commercial street are listed in Appendix C (Table C1). The *post-hoc* Tukey's test is generally used to determine the variability of the meteorological variables across different spaces (40). In this study, the test was applied to confirm that the meteorological variables for the measurement sites in the two study areas differed significantly (Figure 3).

Overall, the meteorological indicators for CP3 in the coastal park were not significantly different from those of CP1 (*T*_*a*_), CP2 (*RH, UTCI*), CP4 (*RH, V*_*a*_, *T*_*g*_, *T*_*mrt*_) and CP5 (*T*_*mrt*_); the meteorological indicators for CP6 (*V*_*a*_) were not significantly different from those of CP1 (*V*_*a*_) and CP5 (*RH, T*_*g*_).

The meteorological indicators of CS3 in the commercial street were not significantly different from those of CS1 (*T*_*g*_, *T*_*mrt*_, *UTCI*), CS2 (*UTCI*), CS4 (*V*_*a*_, *T*_*mrt*_) and CS6 (*V*_*a*_, *T*_*g*_); Measured values in CS1 and CS4, were not significantly different from CS2 (*T*_*g*_), CS5 (*RH*) and CS2 (*T*_*g*_), CS6 (*V*_*a*_); all other measurement points portrayed strong variability.

#### Thermal sensation vote (TSV)

In outdoor spaces, the meteorological variables (*T*_*a*_, *RH, V*_*a*_, and *T*_*g*_) can significantly affect human thermal sensation (72). To quantify the effects of these variables on the TSV, we conducted the Spearman's correlation analysis of meteorological variables and TSV for both study areas and compared the results with other studies (Table 3).

**Table 3:** Spearman's correlation results for thermal sensation vote (TSV) and meteorological parameters of both the study areas.

**City**	**T_a_**	**RH**	***V_*a*_***	**T_g_**
CP (this study)	0.319**	−0.197**	−0.178**	0.381**
CS (this study)	0.329**	−0.236**	−0.060	0.359**
Harbin (19)	0.577**	−0.319**	−0.129**	0.560**
Xi'an (16)	0.63**	−0.55**	−0.20**	0.70 **

^**^Significance at 0.01 level; T_g_, Globe temperature; T_a_, Air temperature; RH, Relative humidity; *V*__*a*__, Wind speed.

In summer, *T*_*g*_ (ρ_coastalpark_ = 0.381, ρ_commercialstreet_ = 0.359) and *T*_*a*_ (ρ_coastalpark_ = 0.319, ρ_commercialstreet_ = 0.329) were considered to be the main factors that influenced the respondents' thermal perceptions, which was consistent with the results of most studies (19, 25, 52, 73). The RH (ρ_coastalpark_ = −0.197, ρ_commercialstreet_ = −0.236) and *V*_*a*_ (ρ_coastalpark_ = −0.178, ρ_commercialstreet_ = −0.060) had negative correlation with the TSV, and for the commercial street, *V*_*a*_ (ρ_commercialstreet_= −0.060) did not show a correlation with the TSV, which could be attributed to the lower wind speed in the urban high-density central area. Compared to those in Harbin and Xi'an, the correlations of *T*_*a*_, *T*_*g*_, RH, and *V*_*a*_ (except for commercial street) to TSV in CP and CS did not differ significantly, indicating that each climate indicator had an impact on the OTC in the coastal city in summer.

**Figure 4:**
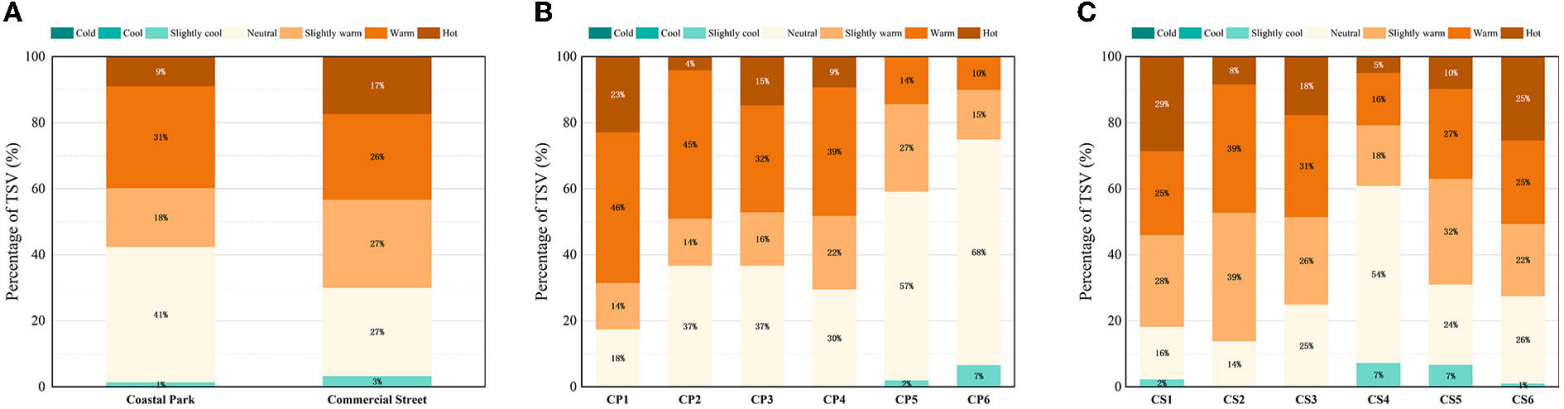
TSV in open spaces during summer: **(A)** Both study areas, **(B)** coastal park, and **(C)** commercial street.

There were some differences between the TSVs of the coastal and commercial areas (Figure 4A). The TSV values tended toward points that indicated a hot perception (TSV > 0), with 70% for the commercial street and 58% for the coastal park. The coastal park had the highest percentage of “neutral” scores (TSV = 0; 41%), followed by “hot” (TSV = 2; 31%). In the commercial street, the proportions of “neutral” (TSV = 0), “slightly warm” (TSV = 1), and “hot” (TSV = 2) scores were about the same (~27%). The proportion of commercial street (17%) considered to be “hot” (TSV = 3) was twice as high as the proportion of the coastal park (9%).

The majority of people (82%) had hot feeling (TSV ≥ 1) and 23% felt “hot” (TSV = 3) at the exposed beach (CP1) in the comparison to the sites in the coastal park (Figure 4B). The TSVs at CP2, CP3, and CP4 were similar, with about 40% choosing “warm” (TSV = 2). The TSV of CP5 and CP6 were considered “neutral” (TSV = 0) by more than half of the respondents, probably because the measurement points were in the shade, and the perceptions of CP6 indicated “neutral” heat (TSV = 0; 68%); moreover, 7% of volunteers chose “slightly cool” (TSV = −1), which indicated that people felt more comfortable in a natural sheltered environment in the coastal zone.

In the commercial street, the proportion of the respondents who felt hot at each measurement point was significantly higher than of those in the coastal park (Figure 4C). More than 80% of the respondents felt warm (TSV ≥ 1), and approximately 50% felt extremely warm (TSV ≥ 2) in the front plazas of CS1 and CS2, with the highest proportion (29%) feeling “hot” (TSV = 3) in CS1. Among the “neutral” (TSV = 0) votes, those for CS3, CS5, and CS6 were accounted for ~25%, but interestingly, the proportion for CS4 was more than half, which may be caused by the proximity of CS4 to high-rise buildings and the fact that the area was in the shade (due to building shadows). Overall, the more open the commercial spaces (CS1 and CS2) were, the hotter the perceived climate, while the more shaded the space (CS4), the more moderate was the perceived climate, which was consistent with the results obtained for the coastal park.

In addition, in the preference poll for meteorological variables, the two regions portrayed consistency (Appendix D), with the respondents preferring lower *T*_*a*_, solar radiation and no change RH, *V*_*a*_. with the factors being more pronounced for the commercial street. Surprisingly, the proportion of preferring stronger wind in commercial streets (32%) less than that in coastal park (36%).

#### Thermal comfort vote (TCV)

**Figure 5:**
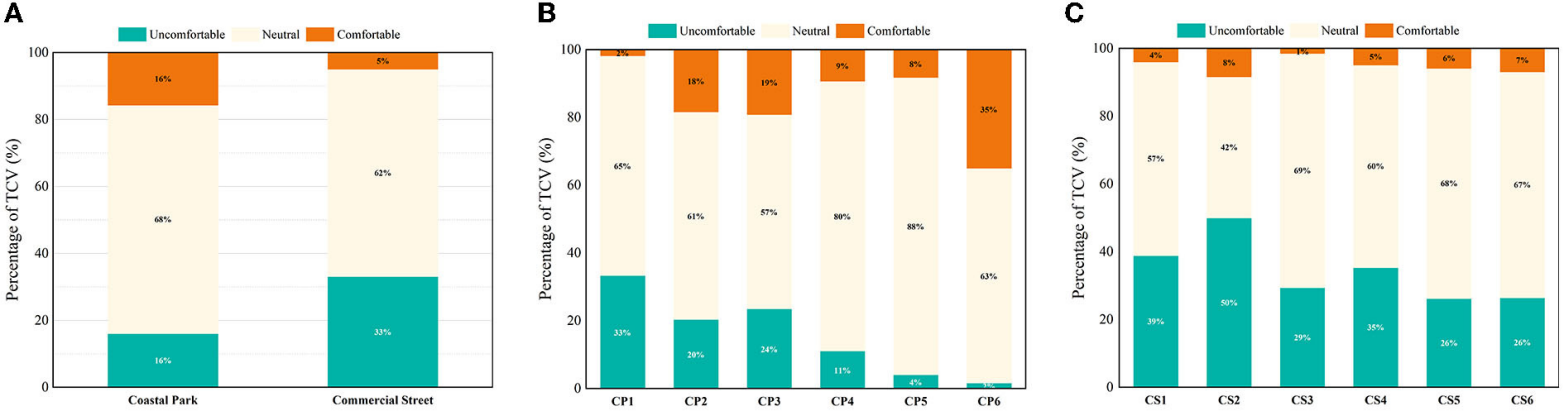
TCV in open spaces during summer: **(A)** Both study areas, **(B)** coastal park, and **(C)** commercial street.

In summer, there were some differences in the thermal comfort vote (TCV) between the coastal park and commercial street (Figure 5A). The proportion of votes that indicated “neutral” climate was about the same in both the zones (around 65%); those indicating “comfortable” climate was same as those that indicated “uncomfortable” climate in the coastal park (16%). However, the proportion of “uncomfortable” votes was much higher than that of “comfortable” votes, at 33%, while “comfortable” votes only accounted for 5% of the total votes. Overall, people in both zones were mostly accepting of the thermal environment, but the coastal park was significantly more comfortable than the commercial street.

In the coastal park, for the CP1 area, most votes tended to indicate a strong “uncomfortable” (33%) climate, with only a few votes indicating a “comfortable” (2%) climate, in contrast to CP6, which portrayed the most votes for “comfortable” (35%) climate and least votes for “uncomfortable” (2%) climate (Figure 5B). The CP4 and CP5 sites had the highest percentage of votes for “neutral” climate, at 80 and 88%, respectively, with the other regions portraying proportions close to 60%. Similar to the heat sensation poll, the two shaded areas, CP5 and CP6, in the coastal zone were the most popular areas.

In the commercial street, more areas were considered to be “uncomfortable,” compared to the coastal park (Figure 5C), with CS2 portraying the highest votes indicating an “uncomfortable” climate (50%), followed by CS1. Surprisingly, for the votes obtained for CS4, more than half of the TSV poll was “neutral” (54%), and 7% considered the climate to be “slightly cool,” with 35% voting “uncomfortable” in the TCV poll. The CS3 site portrayed the most “neutral” rating (69%), while CS5 and CS6 both portrayed the lowest “uncomfortable” rating of 26%. It is possible that both measurement points were in shaded areas (located in the shadow regions of large buildings to the south).

The TCV voting results were consistent with most studies (25, 47, 74), concluding the fact that, in summer, outdoor spaces shaded by trees or buildings were more comfortable than the areas with lower SVF.

**Figure 6:**
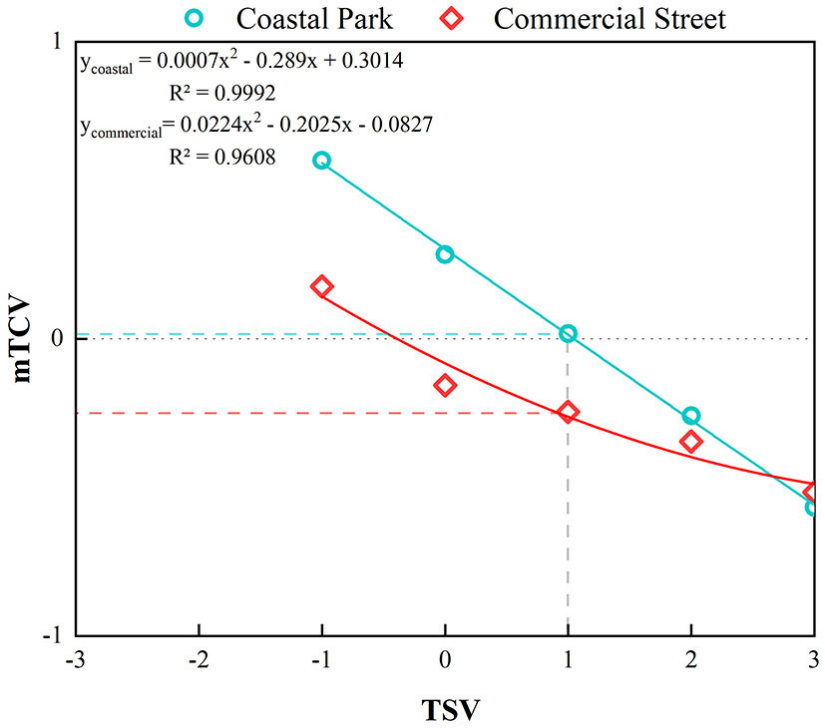
Correlation between TSV and TCV for coastal park and commercial street.

Different TSV levels were matched to the corresponding weighted mean TCV (mTCV) (Figure 6). The coastal park portrayed a strong correlation between the TSV and the thermal comfort of the respondents, i.e., the higher the heat sensation, the more uncomfortable the respondents were. When these respondents felt thermally comfortable (TCV>0) in summer, TSV < 1.05 for coastal park and TSV < −0.47 for commercial street, it reflected the fact that people in the coastal park are more capable of thermal adaptation than those in the commercial street. When the respondents felt “slightly warm” (TSV = 1), the TCV values were 0.01 and −0.26 for the coastal park and commercial street, respectively, in the “slightly warm” condition, respondents in the coastal area felt “neutral” (TCV = 0), while those in the commercial area felt slightly uncomfortable (TCV < 0). The results indicated that psychological factors and environmental factors influenced the comfort level of people in the same feeling of heat.

### Outdoor thermal benchmarks

#### Neutral UTCI (NUTCI) and neutral UTCI range (NUTCIR)

Neutral temperature is defined as the temperature at which people feel neither cold nor hot (75), and it is considered to be a valid indicator for evaluating thermal comfort.

**Figure 7:**
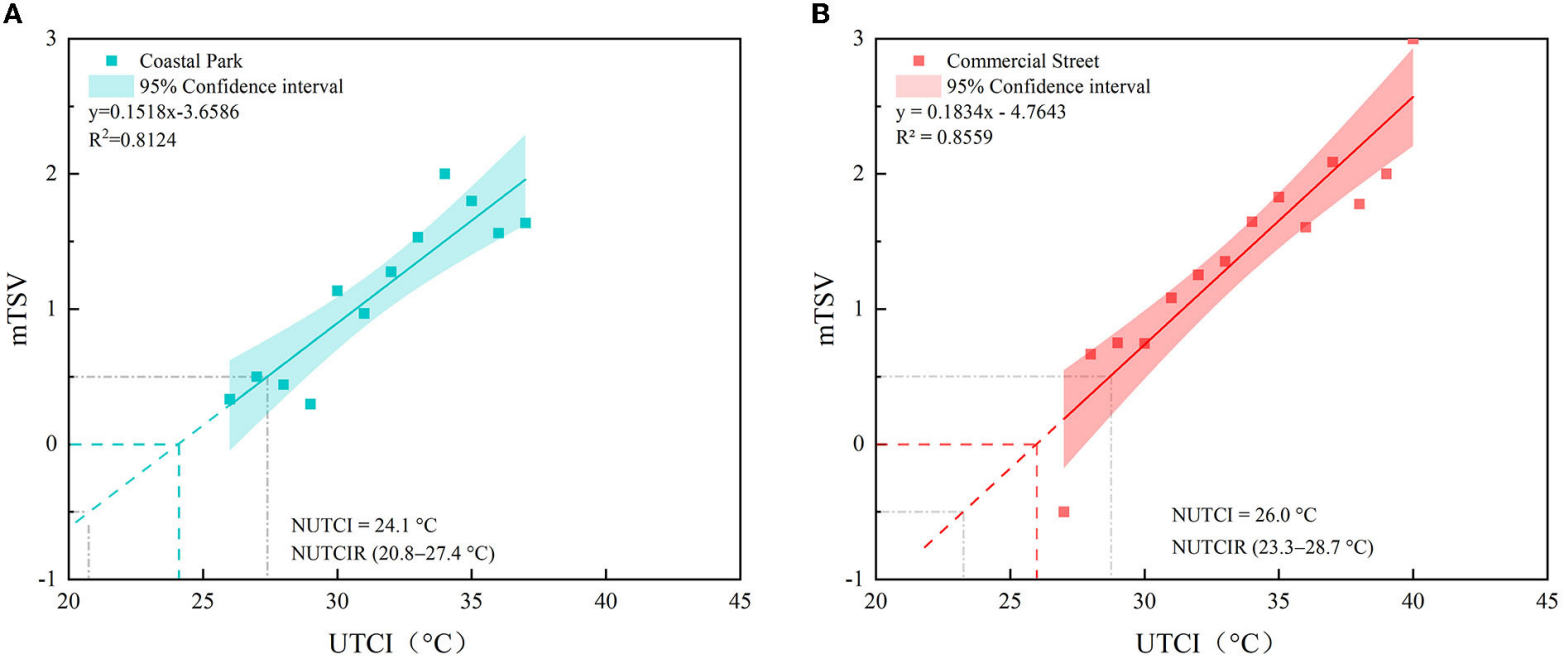
Neutral temperatures calculated using a linear regression: **(A)** Coastal park and **(B)** commercial street.

In this study, we calculated the NUTCIs for the coastal park and commercial street to analyze the difference in the thermal sensation between the waterfront and built-up areas of the coastal city of Dalian. The weighted average of summer TSV and 1°C UTCI was calculated and fitted using linear regression (73). The UTCI was considered to be neutral when mTSV = 0. The NUTCI was significantly higher in the commercial street (26.0°C) than that in the coastal park (24.1°C), and this difference may be closely related to air temperature, clothing thermal resistance, and psychological expectations (44, 76) (Figure 7).

The UTCI range of the mTSV (−0.5, 0.5) was the neutral range (70), which represented the range generally accepted by most people. The NUTCIR of the coastal park (20.8–27.4°C) was slightly wider than that of the commercial street (23.3–28.7°C). The upper and lower limits of the NUTCI and NUTCIR of the coastal park were smaller than those of the commercial street, which indicated that the respondents in the commercial street were more adapted to the high-temperature environment in the high-density built-up area, whereas the reinvents in the coastal park preferred a cooler environment.

#### Thermal acceptable range (TAR)

The thermal acceptable range (TAR) is often used to assess OTC conditions, and the ASHRAE standard 55 defines the TAR as the range of temperatures acceptable to at least 80% (normal conditions) or 90% (strict conditions) of the residents (60). To explain the subjective thermal sensations of the UTCIs at different conditions, it is necessary to define the NUTCIR in which respondents feel comfortable. In this study, we used the range of acceptable temperatures for 80% of the respondents.

**Figure 8:**
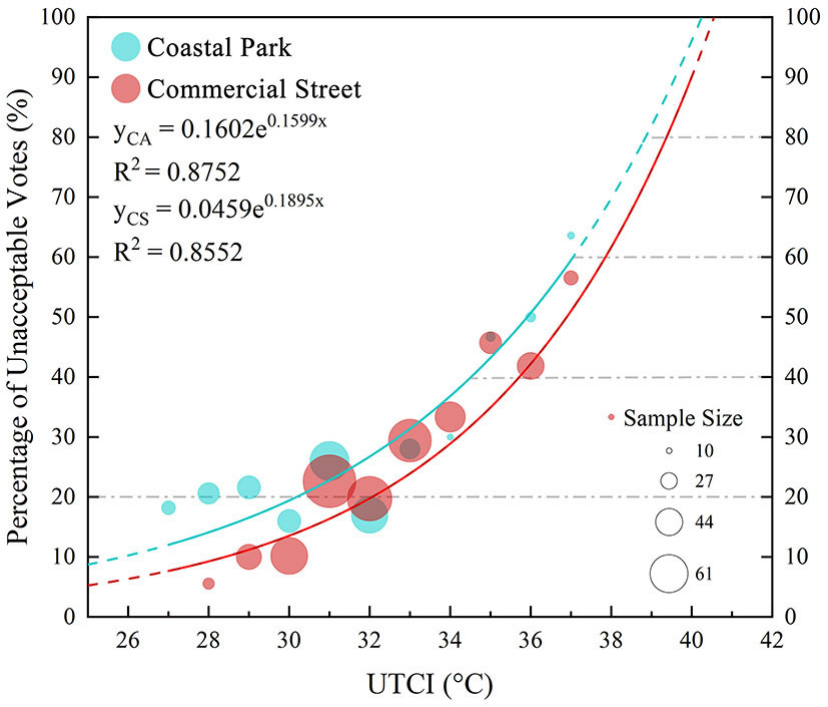
Relationship between the thermal unacceptable rate and UTCI for the coastal park and commercial street.

Since our survey was conducted during hot weather and almost no respondents felt cold, as evidenced by the TSV, this study lacks subjective perceptions related to cold stress (only a limited range of thermal sensations was studied). Therefore, we calculated the thermal unacceptable rate of the UTCI in the 1°C interval, fitted it to an exponential function, and corrected it only for the four thermal stress levels of the UTCI scale, which corresponded to unacceptable levels of 40, 60, 80, and >80% (77, 78) (Table 4). In hot summer weather, the upper limit of the TAR was 32.1°C in the commercial street and 30.2°C in the coastal park (Figure 8).

**Table 4:** UTCI calibrations for different stress categories.

**Thermal stress**	**Modified UTCI** **(coastal park) (°C)**	**Modified UTCI** **(commercial street) (°C)**	**UTCI (°C)**
Extreme heat stress	>38.9	>39.4	>46.0
Very strong heat stress	37.1–38.9	37.9–39.4	38.0–46.0
Strong heat stress	34.4–37.1	35.7–37.9	32.0–38.0
Moderate heat stress	30.2–34.5	32.1–35.7	26.0–32.0
No thermal stress	< 30.2	< 32.1	9.0–26.0

### Thermal adaptations

**Figure 9:**
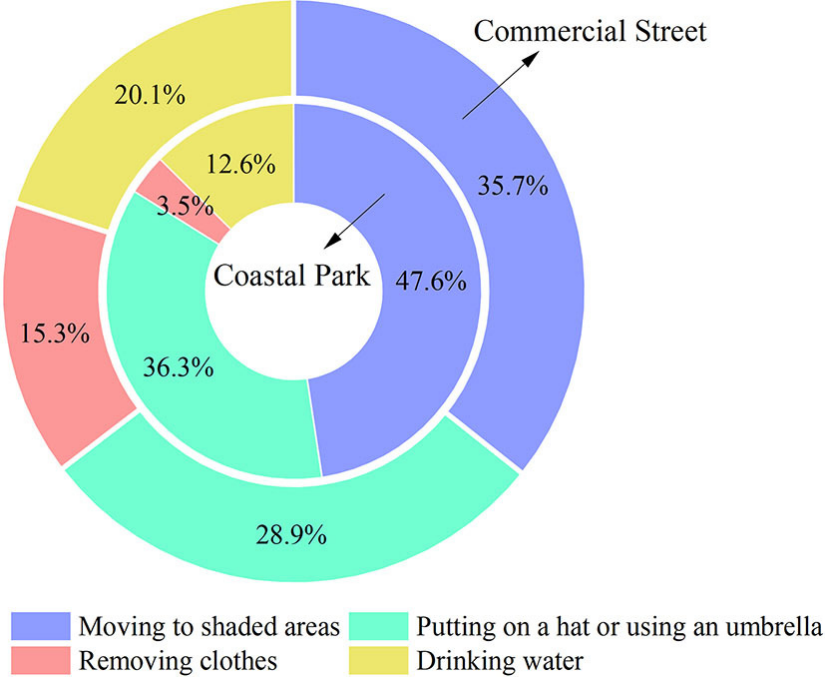
Comparison of preferred thermal adaptation behaviors for both the study areas.

We selected the respondents' subjective opinions and analyzed the differences in their environmental improvements and needs in different spaces (Figure 9). The respondents in the coastal park preferred “moving to shaded areas” to relieve discomfort (47.6%), followed by “putting on a hat or using an umbrella” (36.3%) and “drinking water” (20.1%). The option of “removing clothes” was the least preferred (3.5%) because, in general, people in coastal zones had less thermal clothing resistance (0.22 clo), compared to those in the commercial street (0.32 clo), due to the fact that clothing blocks a majority of ultraviolet rays. The choices of the respondents in the commercial area were more balanced, with the majority choosing “moving to shaded areas” (35.7%) and “putting on a hat or using an umbrella” (28.9%) to relieve discomfort, followed by “drinking water” (20.1%) and “removing clothes” (15.3%). The results indicated that people in both regions preferred to adjust their thermal comfort by moving to shaded areas.

### Thermal environment optimization

**Figure 10:**
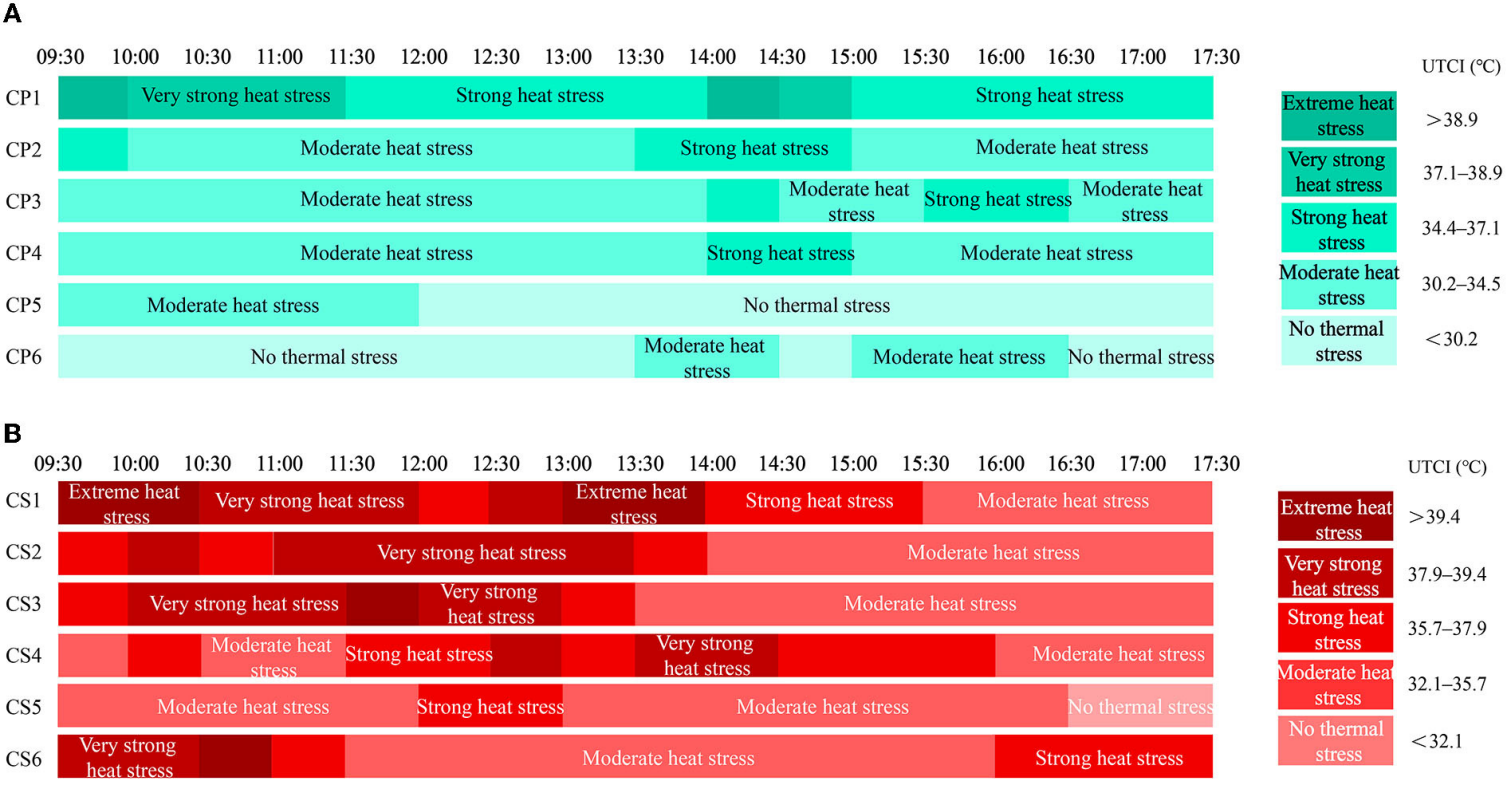
UTCI calendar for the 12 measuring sites considered in this study: **(A)** Coastal park and **(B)** commercial street.

The calendar, combined with the modified thermal benchmark, can visualize the thermal environment conditions and changes in outdoor spaces and provide a reference for viable optimization strategies for thermal environments (75). In this study, we divided the thermal stresses in 30-min intervals for each space in the two regions and used the corresponding thermal stresses of “Modified UTCI_Coastalpark_” and “Modified UTCI_Commercialstreet_” to describe the thermal environment of each space. As shown in Figure 10, each thermal stress corresponds to a different color.

In the coastal park, the shaded spaces (CP5 and CP6) had comfortable thermal conditions most of the time, with “moderate heat stress” experienced only from 09:30 to 12:00 h (CP5), 13:30 to 14:30 h and 15:00 to 16:30 h (CP6), which was related to the orientation of the shade. Semi-open spaces (CP2, CP3, and CP4) were under “moderate heat stress” most of the morning time and “strong heat stress” briefly between 13:30 and 16:30 h. According to our findings, we recommend that seating areas must be set up in the shade around the semi-open space. The beach (CP1) was exposed to sunlight and subjected to “strong heat stress” for extended periods of time; however, most people visit the beach to experience the sun and see the natural scenery, and therefore, simple sun protection devices (e.g., tarps, gazebos, and parasols) can be installed along the beach shoreline. Additionally, during 09:30–11:30 h and 14:00–15:00 h, people should avoid visiting the beach unless adequate protective measures have been taken (Figure 10A).

All spaces in the commercial street experienced thermal discomfort, the thermal conditions of the spaces were not stable, and the conditions at some measurement points were highly variable due to the shades of building and self-shading (Figure 10B). The CS1 site was in “strong heat stress” for a long time and “extreme heat stress” or “very strong heat stress” from 09:30 to 12:00 h and 12:30 to 14:00 h. The CS2 and CS3 sites were in “moderate heat stress” in the afternoon (14:00–17:30 h) as a result of building shadow, and in “strong heat stress” or hotter in the morning and noon (9:30–13:30 h). The CS4 site experienced “strong heat stress” or “very strong heat stress” from 11:30 to 16:00 h but was more comfortable in the morning and evening h. Both CS5 and CS6 sites were located on the north side of the buildings and were in “moderate heat stress” most of the time, but CS5 experienced “strong heat stress” in the afternoon (12:00–13:00 h), while CS6 experienced it or stronger in the morning and evening (09:30–11:30 h, 16:00–17:30 h). The difference was mainly due to the different heights and forms of the south side of the building. In the commercial street, most of the measurement points showed different heat stress in the morning, mid-day and evening due to the differences in openness, street orientation, building height and morphology. Therefore, we recommend planting large-canopy street trees at the curb of south-facing roads to provide shade, along with permeable paving, temporary sprinklers, and shading devices, according to the UTCI calendar. In addition, optimizing the building form and promoting urban ventilation may be a better way to improve the thermal environment of the commercial street (79).

## Discussion

### Neutral UTCI (NUTCI)

In Dalian, during summer, the NUTCI of the coastal park (24.1°C) was significantly lower than that of the commercial street (26.0°C), which corresponded to the difference in the mean UTCI of the two areas (mean UTCI_Coastalpark_ = 31.1°C; mean UTCI_Commercialstreet_ = 33.6°C). In addition, the clothing preference, age and psychological expectation of the respondents may influence individual thermal assessment in different environments; for example, the air temperature was cooler in the coastal park, but the clothing thermal resistance (0.22 clo) was lower than that in the commercial street (0.32 clo), which was because most people who visited the coastal area had increased physical contact with nature (e.g., swimming and experiencing sea breeze). The respondents in commercial district chose clothing with greater thermal resistance to withstand the large temperature difference between the indoor and outdoor environments, while to follow social etiquettes. Also, the proportion of older people, who were less thermally sensitive than younger people, in the coastal park (41.3%) was significantly higher than that in the commercial street (12.4%).

Previous studies have proven that the variations in the NUTCIs of the cities in the same climate zone may be significant, depending on the study scope, method, season, and background climate (80) (Appendix E, Table E1). The findings of the present study were compared to those of the previous studies conducted during the same season and using the same method. Dalian and Harbin are in the same climate zone (Dwa), however, in our study, the NUTCI of the Harbin Commercial Street (19.3°C) (19) was lesser than that of the Dalian Commercial Street (26.0°C). This can be attributed to the fact that Harbin is located in an extreme cold region (whereas Dalian is a cold region), and the average summer temperature in Harbin (21.9°C) is significantly lower than that of Dalian (23.9°C) (27, 56). Additionally, time of test period also has a large impact on the test results (19), Harbin's test time span is larger (2 months), while this study concentrates on the hottest period of summer (2 weeks).

Nagoya in Japan and Umeå in Sweden are coastal cities like Dalian, and in summer, the NUTCI in Umeå Park is only 14.4°C (42), which is nearly 10°C lower than that of the Xinghai Park in Dalian. This may be because Umeå is located in a subarctic region, with short and warm summers, and the locals have adapted to this colder climate. The campus NUTCI in Nagoya was 10°C higher than that in the Xinghai Park in Dalian (47); such a large difference may be directly related to the age, gender, and cultural background of the respondents. First, the volunteers in Nagoya came from a narrower age group (college students) and were all male, whereas the age range of the respondents in this study (12–91 years) was broad. Second, cultural background and thermal environment influenced the individual NUTCI (11); for example, Japanese men still choose heavy suits in extremely hot weather to adhere to social etiquette. Notably, the NUTCI of the Xinghai Park in Dalian was slightly lower than that of the cities, such as Chengdu (24.8°C) (20), Iran (25.8°C) (18), and Guangzhou (26.0°C), that experience hot summers (71). The NUTCI of the coastal park in Dalian was significantly higher than that of children's park in Xi'an (17.8°C) (81), because the study participants in Xi'an were children and had less heat tolerance.

### Neutral UTCI range (NUTCIR)

The UTCI range of MTSV (−0.5, 0.5) is a neutral range (60), which represents a generally acceptable range. In this study, the NUTCIR for the coastal park (20.8–27.4°C) was significantly wider than that for the commercial street (23.3–28.7°C), indicating that the respondents in the marina area were more accepting of the coastal park, which may be due to the fact that the beach area was cooler than the urban center. The upper and lower limits of the NUTCI and NUTCIR for the commercial street were higher than those for the coastal park; therefore, the respondents in the commercial areas were more accustomed to the warmer environment.

Generally, geographic location and local climate characteristics have a strong relationship with the NUTCIR (Appendix E, Table E2). In the park and campus studies, the NUTCIR of the costal park in Dalian was wider, compared to that of the inland cities (Guangzhou, Tehran, and Chengdu), because in Dalian, the variations in the meteorological indicators were more stable. The comparative analysis with other cities indicated that, among the seaside cities considered in this study, the ΔNUTCIR of Umeå (5.7°C) was close to that of Dalian, whereas Nagoya had a small ΔNUTCIR (3.7°C), this may be related to the over-concentration of the respondents' age groups (all respondents were male college students). Hong Kong portrayed a higher and wider NUTCIR than Dalian, owing to that fact that Hong Kong is a high-density city, with extremely hot summers, and the long-term thermal experience and adaptation of the residents has enabled them to adapt to higher temperatures. In the commercial area study, the NUTCIR of the commercial street in Dalian (23.3–28.7°C) was higher than that of Harbin (15.6–23.0°C), due to the higher summer temperatures in Dalian; however, Dalian's ΔNUTCIR (5.4°C) was significantly smaller than that of Harbin (7.4°C), which may be caused by hot summer weather in Dalian during the testing period.

### Thermal acceptable range (TAR)

Table E3 (Appendix E) portrays the results of several OTC studies on TAR. Because our tests were conducted during the hottest time of summer in Dalian and lacked cold stress samples, we could only measure the upper limit of the TAR, similar to a previous study conducted on Guangzhou (71). During the hot summer weather in Dalian, the upper limit of the TAR was 32.1°C in the commercial street and 30.2°C in the coastal park. We compared these results with the thermal comfort study conducted on the Guangzhou campus; the upper limit of the TAR in the campus was between the Dalian coastal park and the commercial street, which was because Guangzhou had a typical subtropical climate, with hot and humid all year round and higher temperatures than Dalian in summer; therefore, the upper limit of the TAR was higher than that of the coastal park in Dalian, while the temperature and the UTCI levels in the commercial street of Dalian during the test period were higher than those in Guangzhou, thus indicating that the upper limit of TAR_Commercialstreet_ was higher.

**Figure 11:**
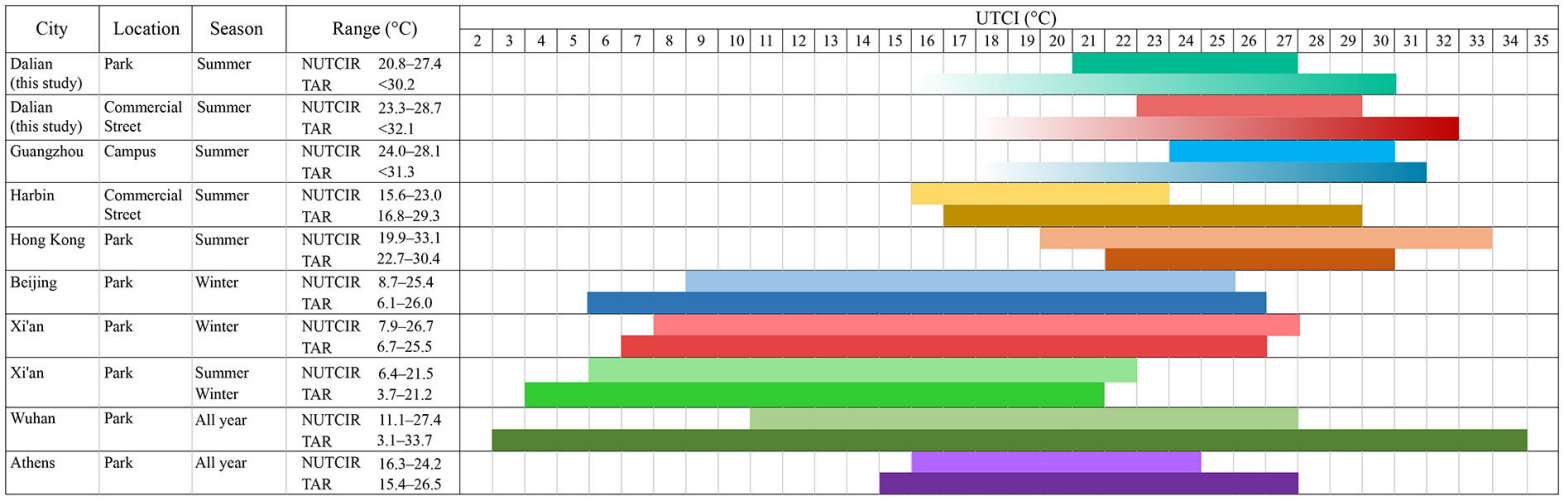
Relationship between NUTCIR and TAR.

Further, we combined the results of the TAR and NUTCIR and discussed the difference between their predicted and actual comfort ranges (Figure 11). In most studies, the difference between the intervals of the TAR and NUTCIR was not significant, such as that observed in studies conducted on Beijing and Xi'an (80). Notably, the upper limits of TAR in the coastal park and commercial street of Dalian were both significantly higher than the upper limit of the NUTCIR, which was similar to the results of the study conducted on Guangzhou (71). We believe that, in the coastal park, although the residents felt hot in summer, the temperature was still acceptable, because most of the respondents actively engaged in outdoor leisure activities such as beach excursions or commercial shopping and had strong psychological expectations for the hot environment. In contrast, Harbin, an inland city with severe cold, portrayed a surprisingly high TAR ceiling of 6°C (19), compared to the NUTCIR, which could be related to Harbin's long and severe winter weather; therefore, the local residents looked forward to the warm weather in summer and thus portrayed extreme high acceptance for warm environments. In Hong Kong, which is also a coastal city, the upper TAR limit was lower than the upper NUTCIR limit (21), which may be due to the long hot summers in Hong Kong and the urban heat island effect caused by high-density construction, resulting in the citizens' non-acceptance of the local thermal environment. This difference in the psychological and physical sensations of the residents can be explained by estimating the complexity of their thermal sensations.

### Implications

The thermal comfort survey of coastal park and commercial street showed that people's thermal perception and adaptive behavior in coping with summer heat reflected consistency, i.e., people preferred shaded areas during summer daytime, the percentage of “comfort” was significantly higher for TSV and TCV in both areas with measurement points in the shade of trees, structures, and buildings, and people were more willing to move to shaded areas to cope with thermal discomfort. In the coastal park, the proportion of feeling comfortable (TCV = 1) in the shade of trees was significantly higher (CP6, 35%) than under the pavilion (CP5, 8%), which indicates that the optimal design of landscape features can improve the OTC of citizens (40). In addition, in the correlation analysis between meteorological variables and TSV in both areas, the wind speed in the coastal area showed a significant correlation, while the commercial street did not, but in the preference votes for meteorological variables, the proportion of “preferring stronger wind” in the coastal area was greater than in the commercial street, probably because the wind speed in the commercial street area was unstable, with high winds and static winds were more frequent. Combining the above discussion and related results, the following five suggestions for urban design and landscape design are proposed: (1) Setting up plant shade in outdoor resting places in the coastal area, and planting more street trees in the commercial area to relieve heat pressure on residents (82); (2) In outdoor open areas, such as beaches and squares, structures such as shade shelters can be set up for shade, which combined with spraying, can cool the temperature and increase humidity; (3) Optimizing the building form layout to increase building shading in open areas to reduce the risk of thermal exposure in high-density areas (83); (4) Providing the public with a thermal calendar for different areas in summer and advice on the risks associated with the thermal environment; (5) Optimizing the wind environment of the pedestrian layer in high-density neighborhoods in combination with sea breeze, and set wind-guiding or wind-blocking measures according to seasonal characteristics (84–86).

### Limitations

Due to the uncontrollable nature of outdoor testing, in the future, our study can be supplemented on an ongoing basis. First, in this study, we considered the thermal comfort in coastal cities only during summers. Compared to that of studies conducted in multiple seasons, the range of the NUTCIs for the studies conducted during summers was smaller (Figure 11), and the discussion of influences and design strategies to promote and extend the outdoor activity period could be continued in the winter and transition seasons while refining the revised UTCI scale. Second, our study focused more on the spatial differences of important metrological factors, and the effects of different levels of natural environmental elements (e.g., vegetation, sea breeze, and white noise) on individual comfort need to be explored in the future. Third, the intensity of pre-test activities could lead to metabolic changes, which could affect individual comfort. For example, the study on Guangzhou was limited to the thermal comfort of students during summer military training (71), and some elderly people in our study at the coastal park engaged in activities of different intensities, such as square dancing, jogging, and swimming, which could be discussed in future studies.

## Conclusions

In this study, we considered two tourist-friendly places, a coastal park (Xinghai Park) and a high-density commercial street (Xi'an Road), in Dalian, China. Six spatial measurement sites were selected in each of the two areas to compare the differences in the thermal environment of outdoor leisure activity sites in a coastal city in summer. The differences in the thermal benchmark and adaptation were compared through meteorological measurements and questionnaires, and the following conclusions were obtained.

(1) During summer, in the coastal city, there was a significant correlation between the TSV and all outdoor thermal ambient meteorological parameters. The coastal park and commercial street portrayed consistent characteristics, with *T*_*a*_ and *T*_*g*_ being positively correlated with the TSV and *V*_*a*_ and RH being negatively correlated with the TSV. Notably, *T*_*g*_ had the strongest correlation with TSV and was the main factor that influenced the OTC in coastal cities. The TSV and TCV results were consistent with other studies, with respondents feeling more comfortable in the shade of buildings or trees and a general uncomfortable feeling in areas with high openness and exposure.
(2) There was a significant difference in the range of thermal comfort between the coastal area and the city center in summers. The NUTCI_Coastalpark_ was 24.1°C and the NUTCIR_Coastalpark_ was 20.8–27.4°C. The NUTCI_Commercialstreet_ was 26.0°C and NUTCIR_Commercialstreet_ was 23.3–28.7°C. This indicated that most respondents in the commercial street were adapted to the high temperatures of high-density built-up areas while those in the coastal area preferred cooler environments and were more adaptable to hot weather.
(3) The TAR results indicated that people had different levels of acceptance to different environments, with an upper limit of 30.2°C in the coastal park and 32.1°C in the commercial street. Notably, unlike previous studies, in our study, the upper limits of the TAR and NUTCIR were significantly different in both the study areas, suggesting that people in coastal cities in colder regions had a high thermal sensitivity to hot weather and strong adaptive capacity.

This study confirmed the applicability of UTCI in summers in coastal areas, explored the subjective satisfaction and objective parameters of thermal comfort in outdoor environments in coastal parks and commercial streets, and analyzed the spatial differences in peoples' thermal comfort evaluation. The results of the study can be used as a criterion to evaluate the OTC in Dalian and provide a reference for designing a comfortable thermal environment in different outdoor spatial environments in the coastal cities in summers. Notably, our study results can improve the overall comfort of different outdoor environments and supplement tourism in coastal area, thereby contributing to the economic development of coastal regions.

## Data availability statement

The original contributions presented in the study are included in the article/Supplementary material, further inquiries can be directed to the corresponding author/s.

## Author contributions

HZ: investigation, data curation, and writing-original draft preparation. FG: conceptualization, methodology, writing-reviewing and editing, and supervision. KL and JW: investigation and resources. JD and PZ: investigation and data curation. All authors contributed to the article and approved the submitted version.

## Funding

This study was supported by the National Natural Science Foundation of China (Nos. 52108044 and 52208045) and the Fundamental Research Funds for the Central Universities (No. DUT21RW204).

## Conflict of interest

The authors declare that the research was conducted in the absence of any commercial or financial relationships that could be construed as a potential conflict of interest.

## Publisher's note

All claims expressed in this article are solely those of the authors and do not necessarily represent those of their affiliated organizations, or those of the publisher, the editors and the reviewers. Any product that may be evaluated in this article, or claim that may be made by its manufacturer, is not guaranteed or endorsed by the publisher.

## Abbreviations

OTC: Outdoor thermal comfort
SVF: Sky view factor
*T*_*g*_: Globe temperature
*T*_*a*_: Air temperature
RH: Relative humidity
*V*_*a*_: Wind speed
UTCI: Universal thermal climate index
*T*_*mrt*_: Mean radiant temperature
ASHRAE: American Society of Heating Refrigerating and Air-conditioning Engineers
TSV: Thermal sensation vote
TPV: Thermal preference vote
TAV: Thermal acceptability vote
PET: Physiological equivalent temperature
TCV: Thermal comfort vote
mTCV: Mean thermal comfort vote
mTSV: Mean thermal sensation vote
NUTCI: Neutral universal thermal climate index
NUTCIR: Neutral universal thermal climate index range
TAR: Thermal acceptable range
LR: Linear regression.
