# Supplementary material for: Spatial differences in thermal comfort in summer in coastal areas: A study on Dalian, China
Source: Front Public Health. 2022 Oct 11;10:1024757. doi: 10.3389/fpubh.2022.1024757 (PMC9592985; doi:10.3389/fpubh.2022.1024757)
Supplement: Supplementary file 1 [file Data_Sheet_1.docx]

# Appendix

## Appendix A: Questionnaire

| 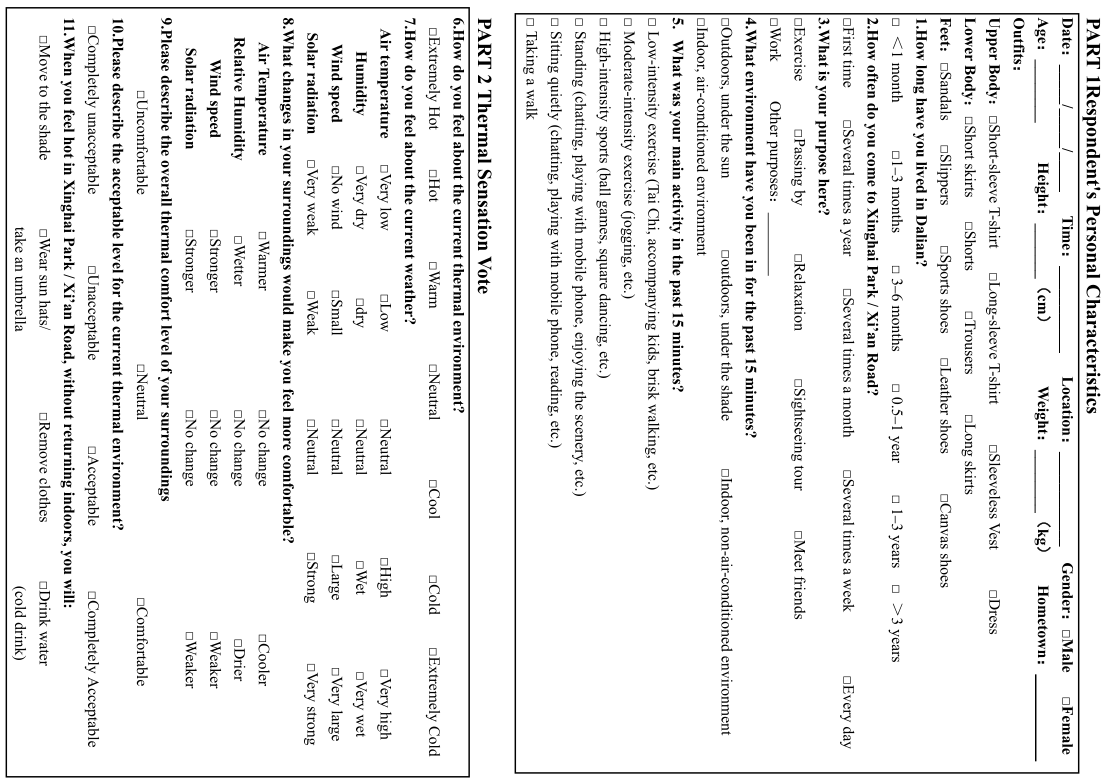 |
| --- |

**Fig. A1.** Thermal comfort questionnaire (translated from the original form written in Chinese)

## Appendix B: Volunteers’ attributes

**Table B1.** Volunteers’ attributes

|  | Coastal Park | Commercial Street |
| --- | --- | --- |
| Gender |  |  |
| Male | 180 (52.8 %) | 262 (49.3 %) |
| Female | 161 (47.2 %) | 269 (50.7 %) |
| Age |  |  |
| <18 | 16 (4.7 %) | 69 (13.0 %) |
| 18–60 | 184 (54.0 %) | 396 (74.6 %) |
| >60 | 141 (41.3 %) | 66 (12.4 %) |
| Height (cm) |  |  |
| MAX | 189 | 190 |
| MIN | 100 | 120 |
| MEAN | 168.6 | 168.6 |
| SD | 9.42 | 8.99 |
| Weight (kg) |  |  |
| MAX | 100 | 130 |
| MIN | 30 | 25 |
| MEAN | 63.6 | 63.7 |
| SD | 11.1 | 13.8 |
| Clothing insulation (clo) |  |  |
| MAX | 0.49 | 0.58 |
| MIN | 0.03 | 0.2 |
| MEAN | 0.22 | 0.32 |
| SD | 0.06 | 0.09 |

*Note: Maximum (Max); Minimum (Min); Standard deviations (SD)

## Appendix C: Meteorological variables

**Table C1.** Meteorological variables in each measured space for both the study areas

|  |  |  | Coastal Park | | | | | | Commercial Street | | | | | | |
| --- | --- | --- | --- | --- | --- | --- | --- | --- | --- | --- | --- | --- | --- | --- | --- |
|  |  | Total | CP1 | CP2 | CP3 | CP4 | CP5 | CP6 | Total | CS1 | CS2 | CS3 | CS4 | CS5 | CS6 |
| T_a_  (°C) | MAX | 33.1 | 33.1 | 31.4 | 31.9 | 29.9 | 29.2 | 28.8 | 36.3 | 35.8 | 35.2 | 35.0 | 36.3 | 33.4 | 36.0 |
|  | MIN | 26.0 | 27.6 | 27.1 | 27.7 | 27.9 | 26.4 | 26.0 | 28.2 | 29.4 | 29.7 | 29.4 | 28.2 | 29.2 | 29.8 |
|  | MEAN | 28.5 | 29.2 | 28.7 | 29.1 | 29.0 | 27.5 | 27.4 | 31.9 | 32.0 | 32.4 | 32.1 | 31.8 | 31.1 | 32.2 |
|  | SD | 1.0 | 1.0 | 0.7 | 0.7 | 0.4 | 0.5 | 0.5 | 1.2 | 1.2 | 1.3 | 0.9 | 1.1 | 0.7 | 1.1 |
| RH (%) | MAX | 87.0 | 87.0 | 82.9 | 83.9 | 82.6 | 85.0 | 85.3 | 72.6 | 72.6 | 68.3 | 64.5 | 67.6 | 68.2 | 66.9 |
|  | MIN | 65.0 | 66.0 | 68.7 | 65.0 | 73.7 | 77.0 | 77.9 | 43.3 | 46.1 | 43.3 | 49.3 | 46.4 | 48.2 | 43.5 |
|  | MEAN | 79.8 | 79.8 | 78.5 | 77.9 | 78.4 | 81.7 | 82.2 | 55.8 | 54.9 | 56.4 | 56.3 | 53.9 | 58.1 | 54.8 |
|  | SD | 3.3 | 4.5 | 2.7 | 3.7 | 1.9 | 1.5 | 1.4 | 4.7 | 5.4 | 4.4 | 2.9 | 3.9 | 4.1 | 4.7 |
| *V_a_* (m/s) | MAX | 3.6 | 1.7 | 3.0 | 2.2 | 2.4 | 3.6 | 2.9 | 5.8 | 5.8 | 2.9 | 5.8 | 4.3 | 5.2 | 4.7 |
|  | MIN | 0.0 | 0.0 | 0.0 | 0.0 | 0.0 | 0.0 | 0.3 | 0.0 | 0.0 | 0.0 | 0.0 | 0.0 | 0.0 | 0.0 |
|  | MEAN | 1.5 | 0.9 | 1.7 | 1.3 | 1.3 | 2.2 | 1.6 | 1.5 | 1.6 | 1.1 | 1.4 | 1.4 | 1.7 | 1.3 |
|  | SD | 0.6 | 0.4 | 0.5 | 0.4 | 0.4 | 0.5 | 0.4 | 0.9 | 1.0 | 0.5 | 0.7 | 0.9 | 0.9 | 0.8 |
| T_g_  (°C) | MAX | 44.7 | 44.7 | 37.6 | 34.3 | 37.4 | 37.4 | 32.1 | 46.0 | 46.0 | 43.6 | 45.3 | 44.3 | 41.3 | 43.4 |
|  | MIN | 27.0 | 34.6 | 28.8 | 28.3 | 29.3 | 27.3 | 27.0 | 29.7 | 30.6 | 30.2 | 33.8 | 30.3 | 29.7 | 31.1 |
|  | MEAN | 33.0 | 40.9 | 34.6 | 31.5 | 31.3 | 30.8 | 28.9 | 36.1 | 39.0 | 37.0 | 38.6 | 37.7 | 32.4 | 34.5 |
|  | SD | 4.3 | 2.2 | 1.6 | 1.6 | 1.2 | 2.6 | 1.1 | 4.6 | 4.5 | 4.6 | 3.7 | 5.1 | 2.2 | 3.3 |
| *T_mrt_* (°C) | MAX | 50.7 | 50.7 | 44.7 | 37.1 | 41.8 | 43.0 | 35.1 | 61.0 | 61.0 | 55.3 | 56.2 | 59.6 | 50.9 | 52.8 |
|  | MIN | 27.1 | 35.8 | 29.3 | 27.5 | 29.7 | 27.3 | 27.1 | 27.6 | 30.9 | 30.3 | 33.8 | 27.6 | 29.7 | 29.6 |
|  | MEAN | 35.4 | 45.6 | 38.3 | 32.7 | 32.5 | 33.2 | 29.8 | 39.4 | 44.4 | 40.1 | 43.4 | 42.3 | 33.6 | 36.2 |
|  | SD | 6.0 | 3.3 | 2.6 | 2.5 | 1.8 | 4.4 | 1.6 | 7.6 | 7.4 | 7.0 | 5.8 | 9.3 | 3.7 | 5.2 |
| UTCI  (°C) | MAX | 38.9 | 39.0 | 35.6 | 34.2 | 34.3 | 33.0 | 31.2 | 40.0 | 40.0 | 39.3 | 39.6 | 38.4 | 36.5 | 39.7 |
|  | MIN | 25.7 | 32.6 | 28.9 | 28.3 | 28.4 | 25.7 | 26.2 | 27.5 | 27.5 | 28.6 | 29.9 | 28.9 | 27.5 | 28.1 |
|  | MEAN | 31.1 | 35.4 | 31.6 | 31.5 | 31.3 | 28.3 | 28.6 | 33.6 | 34.6 | 34.9 | 34.9 | 34.0 | 31.3 | 33.2 |
|  | SD | 2.6 | 1.1 | 1.1 | 1.2 | 0.9 | 1.3 | 0.9 | 2.6 | 2.7 | 2.6 | 2.1 | 2.3 | 1.7 | 2.1 |

*Note: Maximum (Max); Minimum (Min); Standard deviations (SD); Globe temperature (*T_g_*); Air temperature (*T_a_*); Relative humidity (RH); Wind speed (*V_a_*); Mean radiant temperature (*T_mrt_*); Universal thermal climate index (UTCI)

## Appendix D: Preference votes


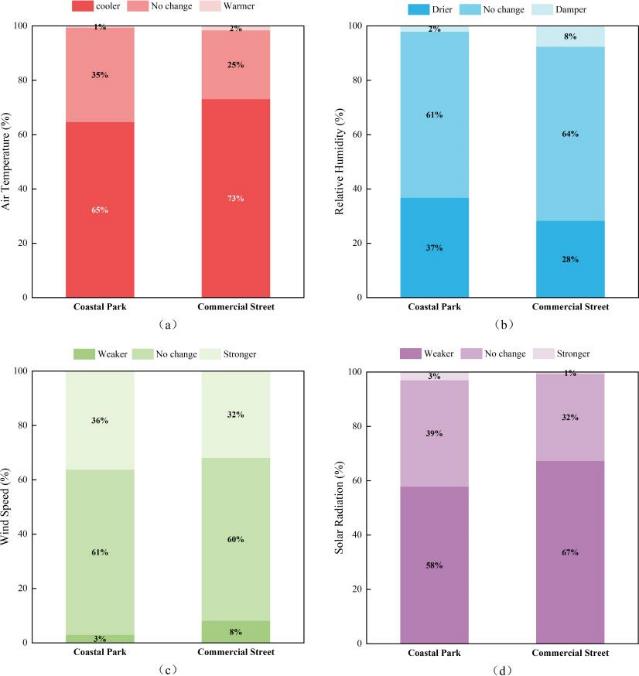


**Fig. D1.** Preference votes for meteorological variables: (a) air temperature, (b) relative humidity, (c) wind speed, and (d) solar radiation

## Appendix E: NUTCI, NUTCIR and TAR for different climate zones and cities

**Table E1.** NUTCI for different climate zones and cities

| City, country | Climate zone | Seasons | Location | Population | NUTCI (°C) | Analysis methods |
| --- | --- | --- | --- | --- | --- | --- |
| Dalian, China (this study) | Dwa | Summer | Commercial Street | Mixed | 26.0 | LR MTSV vs UTCI bin (1 °C) |
| Harbin, China [19] | Dwa | Summer | Commercial Street | Mixed | 19.3 | LR MTSV vs UTCI bin (1 °C) |
| Dalian, China (this study) | Dwa | Summer | Coastal Park | Mixed | 24.1 | LR MTSV vs UTCI bin (1 °C) |
| Nagoya, Japan [47] | Cfa | Summer | Campus | Youth | 34.1 | LR MTSV vs UTCI bin (1 °C) |
| Xi'an, China [82] | Cwa/BSk | Summer | Park | Children | 17.8 | LR MTSV vs UTCI bin (1 °C) |
| Umeå, Sweden [42] | Dfc | Summer | Park | Mixed | 14.4 | LR MTSV vs UTCI bin (1 °C) |
| Guangzhou, China [71] | Cfa | Summer | Campus | Youth | 26.0 | LR MTSV vs UTCI bin (1 °C) |
| Tehran, Iran [18] | BWk | Summer | Campus | Youth | 25.8 | LR MTSV vs UTCI bin (1 °C) |
| Hong Kong, China [21] | Cwa | Summer | Park | Mixed | 22.7 | LR MTSV vs UTCI bin (1 °C) |
| Chengdu, China [20] | Cfa | Summer | Park | Mixed | 24.8 | LR MTSV vs UTCI bin (1 °C) |

Note: Mean thermal sensation vote (MTSV); Universal thermal climate index (UTCI); Linear regression (LR)

**Table E2.** NUTCIRs of different cities and spaces in different climatic zones

| City, Country | Climate zone | Seasons | Location | Population | NUTCIR (°C) | △NUTCIR (°C) | Analysis methods |
| --- | --- | --- | --- | --- | --- | --- | --- |
| Dalian, China (this study) | Dwa | Summer | Commercial Street | Mixed | 23.3–28.7 | 5.4 | LR MTSV vs UTCI bin (1 °C) |
| Harbin, China [19] | Dwa | Summer | Commercial Street | Mixed | 15.6–23.0 | 7.4 | LR MTSV vs UTCI bin (1 °C) |
| Dalian, China (this study) | Dwa | Summer | Coastal Park | Mixed | 20.8–27.4 | 6.6 | LR MTSV vs UTCI bin (1 °C) |
| Nagoya, Japan [47] | Cfa | Summer | Campus | Youth | 32.2–35.9 | 3.7 | LR MTSV vs UTCI bin (1 °C) |
| Umeå, Sweden [42] | Dfc | Summer | Park | Mixed | 11.5–17.2 | 5.7 | LR MTSV vs UTCI bin (1 °C) |
| Guangzhou, China [71] | Cfa | Summer | Campus | Youth | 24.0–28.1 | 4.1 | LR MTSV vs UTCI bin (1 °C) |
| Tehran, Iran [18] | BWk | Summer | Campus | Youth | 23.5–28.1 | 4.6 | LR MTSV vs UTCI bin (1 °C) |
| Hong Kong, China [21] | Cwa | Summer | Park | Mixed | 19.9–33.1 | 13.2 | LR MTSV vs UTCI bin (1 °C) |
| Chengdu, China [20] | Cfa | Summer | Park | Mixed | 22.0–27.5 | 5.5 | LR MTSV vs UTCI bin (1 °C) |

Note: Mean thermal sensation vote (MTSV); Universal thermal climate index (UTCI); Linear regression (LR)

**Table E3.** TAR in different climatic zones

| City, Country | Climate zone | Seasons | Location | Population | TAR (°C) | Analysis methods |
| --- | --- | --- | --- | --- | --- | --- |
| Dalian, China (this study) | Dwa | Summer | Coastal Park | Mixed | ＜30.2 | Thermal acceptable range = 80 % |
| Dalian, China (this study) | Dwa | Summer | Commercial Street | Mixed | ＜32.1 | Thermal acceptable range = 80 % |
| Guangzhou, China [71] | Cfa | Summer | Campus | Youth | ＜31.3 | Thermal acceptable range = 80 % |
| Harbin, China [19] | Dwa | Summer | Commercial Street | Mixed | 16.8–29.3 | Thermal acceptable range = 80 % |
| Hong Kong, China [21] | Cwa | Summer | Park | Mixed | 22.7–30.4 | 1–h acceptable temperature range |
| Beijing, China [80] | Dwa | Winter | Park | Mixed | 6.1–26.0 | Thermal acceptable range = 80 % |
| Xi'an, China [25] | Cwa/BSk | Summer/Winter | Park | Mixed | 12.5–33.7 | Thermal acceptable range = 80 % |
| Tianjin, China [73] | Dwa/BSk | Summer/Autumn/ Winter | Park | Mixed | 12.0–25.0 | LR mTSV vs UTCI bin (1 °C) |
| Chengdu, China [20] | Cfa | Summer, winter | Park | Mixed | 8.1– 28.3 | Thermal acceptable range = 80 % |
| Wuhan, China [26] | Cfa | All year | Park | Mixed | 3.1–33.7 | Thermal acceptable range = 80 % |

Note: Mean thermal sensation vote (mTSV); Universal thermal climate index (UTCI); Linear regression (LR)
